# Air Pollution and Percent Emphysema Identified by Computed Tomography in the Multi-Ethnic Study of Atherosclerosis

**DOI:** 10.1289/ehp.1307951

**Published:** 2014-10-10

**Authors:** Sara D. Adar, Joel D. Kaufman, Ana V. Diez-Roux, Eric A. Hoffman, Jennifer D’Souza, Karen H. Stukovsky, Stephen S. Rich, Jerome I. Rotter, Xiuqing Guo, Leslie J. Raffel, Paul D. Sampson, Assaf P. Oron, Trivellore Raghunathan, R. Graham Barr

**Affiliations:** 1 Department of Epidemiology, University of Michigan, Ann Arbor, Michigan, USA; 2 Department of Environmental and Occupational Health Sciences,; 3 Department of Epidemiology, and; 4 Department of Medicine, University of Washington, Seattle, Washington, USA; 5 Department of Radiology, University of Iowa, Iowa City, Iowa, USA; 6 Department of Biostatistics, University of Washington, Seattle, Washington, USA; 7 Center for Public Health Genomics, University of Virginia, Charlottesville, Virginia, USA; 8 Institute for Translational Genomics and Population Sciences, Los Angeles BioMedical Research Institute at Harbor-UCLA Medical Center, Torrance, California, USA; 9 Medical Genetics Institute, Cedars-Sinai Medical Center, Los Angeles, California, USA; 10 Department of Statistics, University of Washington, Seattle, Washington, USA; 11 Core for Biomedical Studies, Seattle Children’s Research Institute, Seattle, Washington, USA; 12 Department of Biostatistics, University of Michigan, Ann Arbor, Michigan, USA; 13 Department of Medicine, and; 14 Department of Epidemiology, Columbia University Medical Center, New York, New York, USA

## Abstract

Background: Air pollution is linked to low lung function and to respiratory events, yet little is known of associations with lung structure.

Objectives: We examined associations of particulate matter (PM_2.5,_ PM_10_) and nitrogen oxides (NO_x_) with percent emphysema-like lung on computed tomography (CT).

Methods: The Multi-Ethnic Study of Atherosclerosis (MESA) recruited participants (45–84 years of age) in six U.S. states. Percent emphysema was defined as lung regions < –910 Hounsfield Units on cardiac CT scans acquired following a highly standardized protocol. Spirometry was also conducted on a subset. Individual-level 1- and 20-year average air pollution exposures were estimated using spatiotemporal models that included cohort-specific measurements. Multivariable regression was conducted to adjust for traditional risk factors and study location.

Results: Among 6,515 participants, we found evidence of an association between percent emphysema and long-term pollution concentrations in an analysis leveraging between-city exposure contrasts. Higher concentrations of PM_2.5_ (5 μg/m^3^) and NO_x_ (25 ppb) over the previous year were associated with 0.6 (95% CI: 0.1, 1.2%) and 0.5 (95% CI: 0.1, 0.9%) higher average percent emphysema, respectively. However, after adjustment for study site the associations were –0.6% (95% CI: –1.5, 0.3%) for PM_2.5_ and –0.5% (95% CI: –1.1, 0.02%) for NO_x_. Lower lung function measures (FEV_1_ and FVC) were associated with higher PM_2.5_ and NO_x_ levels in 3,791 participants before and after adjustment for study site, though most associations were not statistically significant.

Conclusions: Associations between ambient air pollution and percentage of emphysema-like lung were inconclusive in this cross-sectional study, thus longitudinal analyses may better clarify these associations with percent emphysema.

Citation: Adar SD, Kaufman JD, Diez-Roux AV, Hoffman EA, D’Souza J, Stukovsky KH, Rich SS, Rotter JI, Guo X, Raffel LJ, Sampson PD, Oron AP, Raghunathan T, Barr RG. 2015. Air pollution and percent emphysema identified by computed tomography in the Multi-Ethnic Study of Atherosclerosis. Environ Health Perspect 123:144–151; http://dx.doi.org/10.1289/ehp.1307951

## Introduction

Chronic obstructive pulmonary disease (COPD) is one of the 10 most debilitating illnesses worldwide (Vos et al. 2012). In 2010, 329 million people were estimated to have COPD, with nearly 29,000 productive person-years lost each year. Recent estimates suggest that COPD is currently the world’s third leading cause of death and the fifth leading cause of years lived with disability (Lozano et al. 2013; Vos et al. 2012).

COPD is defined physiologically by airflow limitation that is not fully reversible (Celli et al. 2004; Vestbo et al. 2013). Pulmonary emphysema is defined anatomically by destruction of interalveolar septae and loss of lung tissue and overlaps only partially with COPD. Although smoking is a leading cause of emphysema (Hogg 2004), only weak associations have been documented between emphysema severity and pack-years of cigarette smoking in the general population and in COPD patients (Hogg et al. 1994; Powell et al. 2013). In addition, emphysema has been shown to also develop in never-smokers (Auerbach et al. 1972). Thus, questions remain as to risk factors for the etiology of emphysema.

Exposures to airborne particulate matter (PM) in outdoor, indoor, and workplace air may contribute to the development of emphysema. Epidemiological studies have consistently linked short-term peaks of PM with respiratory outcomes including morbidity and mortality of individuals with COPD (Kelly and Fussell 2011). Greater long-term exposures to air pollution have also been associated with slowed lung growth in children (Avol et al. 2001; Gauderman et al. 2004; Rojas-Martinez et al. 2007) and more rapid decline in lung function in adults (Detels et al. 1991; Downs et al. 2007; Tashkin et al. 1994). Studies have similarly shown that greater long-term levels of PM and traffic-related air pollution are associated with higher incident and prevalent COPD (Andersen et al. 2011; Chen et al. 2005; Karakatsani et al. 2003; Lindgren et al. 2009; Schikowski et al. 2005; Sunyer 2001). To our knowledge, however, there has been no direct assessment of the relationship of ambient air pollution to pulmonary emphysema in an epidemiologic study.

Computed tomography (CT) provides an opportunity to assess pulmonary emphysema and changes in lung structure *in vivo* even among those with normal lung function.(Sanders et al. 1988). Here we examine the associations between long-term exposure to airborne PM ≤ 2.5 and ≤ 10 μm in aerodynamic diameter (PM_2.5_, PM_10_) and oxides of nitrogen (NO_x_; an indicator of traffic pollution) with emphysema-like lung on CT in a large, multi-ethnic cohort of adults. In secondary analyses, we also assessed associations with lung function.

## Methods

*Study sample*. The Multi-Ethnic Study of Atherosclerosis (MESA) recruited 6,814 white, black, Hispanic, and Chinese men and women in Baltimore, Maryland; Chicago, Illinois; Forsyth County, North Carolina; Los Angeles County, California; Northern Manhattan, New York; and St. Paul, Minnesota, between 2000 and 2002 (Bild et al. 2002). Participants, 45–84 years of age, were free of clinical cardiovascular disease at baseline. The MESA Air ancillary study recruited 257 additional participants from Rockland County, New York, and Los Angeles and Riverside Counties, California, in 2006–2007 using the same inclusion criteria (Kaufman et al. 2012). The MESA Family ancillary study recruited 1,542 additional black and Hispanic participants at all MESA centers in 2004–2007. Institutional review board approval and informed participant consent were obtained. Participants without consent for address geocoding and those without complete outcome, exposure, and key covariate data were excluded from statistical analysis.

*Emphysema-like lung (percent emphysema)*. Two sequential axial scans were collected during each participant’s baseline visit using a highly standardized protocol following breath-holds at full inspiration (Carr et al. 2005). Cardiac scans were collected using a multidetector or electron-beam CT, depending on the technology available at each study site, and included approximately 70% of the lung volume from the carina to the lung bases. As described previously (Guo et al. 2002), percent emphysema was quantified by one of several blinded image analysts at a central reading center using the Pulmonary Analysis Software Suite (Guo et al. 2008), which was modified to read the lung fields of a cardiac CT. This measure of emphysema relies on image brightness, which can be used to differentiate tissue from air. Given past pathology research and the mild degree of emphysema in this population, we *a priori* defined percent emphysema as the number of voxels less than –910 Hounsfield Units (HU) divided by the total number of voxels in the lung field (Coxson et al. 1995; Gevenois et al. 1995). Sensitivity analyses explored a –950 HU threshold, which reflects more severe emphysema-like lung regions.

All measures were calibrated using the observed attenuation of air surrounding the body versus a theoretical attenuation of –1,000 HU. Scans with the largest air volume were selected unless there were image quality issues, in which case the higher-quality scan was selected (Hoffman et al. 2009). In a study of 119 participants, excellent agreement for percent emphysema was documented on replicate scans [intraclass correlation coefficient (ICC): 0.89–0.93 at follow-up exams and baseline exams, respectively]. Paired measurements from 10 individuals who were sequentially scanned using both multi-detector and electron beam CTs also demonstrated high correlation (*r* = 0.94) and very small mean differences (< 1%). Finally, validation of 24 individuals with cardiac CT and full lung scans using multi-detector scanners also demonstrated excellent agreement for percent emphysema (ρ = 0.93) (Hoffman et al. 2009).

*Lung function*. Between 2004 and 2007 spirometry was performed on a subset of MESA (*n* = 3,835) and MESA Family (*n* = 92) participants, and on all MESA Air participants (*n* = 257). Participants were randomly selected for spirometry in MESA if they had consented to genetic analysis and had baseline measures of endothelial function; Chinese Americans were also oversampled to ensure adequate sample size for stratified and adjusted analyses (Rodriguez et al. 2010). Spirometry was conducted in accordance with the American Thoracic Society/European Respiratory Society guidelines (Miller et al. 2005) using a dry-rolling seal spirometer (Occupational Marketing, Inc., Houston, TX), and all tests were read by one investigator (Hankinson et al. 2010). Replicate testing of 10% of study participants within 2 weeks of the same examination yielded an average inter- and intratechnician ICC for forced expiratory volume in 1 sec (FEV_1_) and forced vital capacity (FVC) of 0.99. Airflow limitation was defined as having an FEV_1_/FVC and FEV_1_ less than the lower limit of normal (LLN) with a sensitivity analysis definition of only the FEV_1_/FVC ratio less than the LLN (Gläser et al. 2010). LLN were defined using reference equations from the National Health and Nutrition Examination Survey III (Hankinson et al. 1999; Miller et al. 2005) with a 0.88 correction for Asians (Hankinson et al. 2010).

*Participant characteristics*. Participant health data were collected during each examination, including anthropometry measures such as height and weight as well as self-reported information on demographics, medical history, medication use, and smoking exposures (Bild et al. 2002). Urinary cotinine levels were also measured on participants with spirometry. Residential addresses were assigned geographic coordinates using ArcGIS v9.1 (ESRI, Redlands, CA) and the Dynamap 2000 street network (TeleAtlas, Boston, MA).

*Exposure assignment*. Long-term ambient air pollution concentrations were estimated for all participant addresses using residential history data and area-specific prediction models that incorporated time-varying trends and spatial effects using a large suite of spatial covariates detailed elsewhere (Cohen et al. 2009; Raghunathan et al. 2006; Sampson et al. 2011; Szpiro et al. 2010). Our main analyses used modeled-based estimates of average PM_2.5_ and NO_x_ concentrations at participants’ residences during the year before the baseline exam, which were estimated using intensive MESA-specific measurements as well as more spatially limited data from the U.S. Environmental Protection Agency’s Air Quality System (AQS; http://www.epa.gov/ttn/airs/airsaqs/). Because these estimates were not available before 1999, we used these 1-year average exposure estimates as proxies of long-term exposures. We also estimated associations between outcomes and average PM_2.5_ and PM_10_ concentrations between 1980 and 2000 (referred to as 20-year average exposures) that were estimated in a prior MESA ancillary study using models constructed on AQS data for PM_10_ and a PM_2.5_/PM_10_ ratio (Raghunathan et al. 2006). These estimates had more temporal but less spatial information, so they were explored in secondary analyses. For sensitivity analyses we also obtained PM_2.5_ concentrations at AQS monitoring stations and meteorological data from the National Oceanic and Atmospheric Administration (http://www.ncdc.noaa.gov) on the day before each clinical exam.

*Data analysis*. Multivariable regression modeling was performed with SAS v9.2 (SAS Institute Inc., Cary, NC) to examine cross-sectional associations between percent emphysema and long-term exposures to air pollutants. Percent emphysema had a strongly skewed distribution, but because alternate distributions (e.g., the gamma distribution) generated results with similar directionality and significance to our main findings (data not shown), we modeled the outcome as an untransformed variable. Linear regression was used for FEV_1_, FVC, and the ratio of FEV_1_/FVC, and logistic regression was used for airflow limitation (present versus absent).

Modeling was performed with increasing levels of control for potential confounders defined at the time of the examination. All models were adjusted for continuous age and height (with a linear term for percent emphysema models and square terms for pulmonary function models), body mass index (with squared and cubic terms for percent emphysema models and a linear term for pulmonary function models), and pollution as a linear term. Categorical variables in all models included sex, race/ethnicity (white, black, Chinese American, Hispanic), education (< high school, high school degree, some college without a degree, technical or associates degree, bachelors degree, advanced degree), birth location (United States, Puerto Rico, other country), smoking status (never, former, current), pack-years (0, > 0 to 10, > 10 to 20, > 20), cigarettes per day (0 to < 5, 5 to < 10, 10 to < 20, > 20), and exposure to active or secondhand smoke (yes or no). Models for percent emphysema also included a categorical term for CT scanner (electron-beam, non-Siemens multidetector, Siemens multidetector) and an interaction between body weight (≤ 220 lb or > 220 lb) and CT scanner since the radiation was increased 25% for individuals > 220 lb. For our lung function and airflow limitation models, we also controlled for household size and MESA examination (2000–2002, 2004–2005, 2005–2007) and binary variables for hay fever, secondhand smoke exposures (ever or never) in childhood, the workplace, and at home as well as workplace exposures to dust, fumes, or vapors (ever or never). These data (i.e., hay fever, childhood and workplace exposures) were incomplete in the larger cohort, but sensitivity analyses indicated that adjustment did not influence associations between air pollution and percent emphysema. Associations between air pollutants and all outcomes were also robust to adjustment for 1-day average PM_2.5_ concentrations, temperature, and relatively humidity, personal wealth, neighborhood socioeconomic status, asthma before 45 years of age, family history of emphysema, cotinine, cigar and pipe smoking, medication use (i.e., anticholinergics, beta2-agonists, and inhaled steroids), so these covariates were not included in our models in the interest of parsimony. All analyses were controlled for metropolitan area as a fixed effect in the final model to explore potential confounding by study location, though this was expected to reduce power because between-center differences in pollutant levels were known to be large. Mixed models with random effects for site and generalized estimating equations with robust standard errors were also tested in sensitivity analysis but were not presented because they had similar conclusions with respect to direction, magnitude, and significance of the associations and are less able to reliably estimate between-site variability with only six study sites.

Modification of the associations by age (categorized by decade of age), race/ethnicity, sex, education, smoking status, and metropolitan area was also explored using interaction terms and global *F*-tests. Statistical significance was defined based on a *p*-value < 0.05. We furthermore tested the sensitivity of our results to restriction to nonmovers (> 10 years of residential stability).

## Results

Of the 7,014 participants with percent emphysema assessments who consented to geocoding, 6,515 had complete 1-year average exposure and covariate information. Because 20-year estimates of PM_10_ and PM_2.5_ were available in the main MESA cohort only, we investigated these exposures among 4,813 participants. For lung function, we included 3,791 of the 4,182 participants who consented to geocoding based on complete 1-year average exposure and covariate information. Of those, 2,811 had 20-year exposure estimates. For detailed counts of individuals for each analysis, see Supplemental Material, Figure S1.

As shown in Table 1, there were roughly equal numbers of male and female participants with a mean age of 62 years at the time of CT scanning. Approximately 50% were former or current smokers, and 30% had smoked > 10 pack-years. The mean percent emphysema (–910 HU) was 20%. Average percent predicted was approximately 94% for FEV_1_ and 95% for FVC. Approximately 6% of the cohort had airflow limitation by either definition considered. Those included in the secondary analyses of the 20-year exposures were generally similar to those in the primary cohort (Table 1).

**Table 1:** Descriptive characteristics (mean ± SD or %) of study participants.

Characteristic	Emphysema cohort		Lung function cohort	
	1-year estimate(*n*= 6,515)	20-year estimate(*n *= 4,813)	1-year estimate(*n *= 3,791)	20-year estimate(*n *= 2,811)
Percent emphysema (%), –910 HU	19.9 ± 13.4	20.5 ± 13.6	20.2 ± 13.3	20.7 ± 13.5
Airflow limitation (%)^*a*^	5.7	5.8	5.9	5.9
Percent predicted FEV_1_	93.9 ± 17.8	93.5 ± 18.1	93.8 ± 17.9	93.4 ± 18.2
Percent predicted FVC	95.5 ± 16.2	95.2 ± 16.3	95.4 ± 16.2	95.2 ± 16.3
Percent predicted FEV_1_/FVC	98.5 ± 10.7	98.4 ± 10.9	98.4 ± 10.7	98.3 ± 10.9
FEV_1_ (L)	2.4 ± 0.7	2.4 ± 0.7	2.4 ± 0.7	2.4 ± 0.7
FVC (L)	3.2 ± 1.0	3.2 ± 1.0	3.2 ± 1.0	3.2 ± 1.0
FEV_1_/FVC (%)	75.1 ± 8.5	75.0 ± 8.7	75.0 ± 8.5	74.9 ± 8.6
Age (years)	62 ± 10	62 ± 10	61 ± 10	62 ± 10
Female (%)	54	53	51	50
Race/ethnicity (%)
White	37	43	36	39
Black	28	30	24	28
Chinese	11	7	15	10
Hispanic	24	21	25	22
Education (%)
Less than high school	17	15	18	15
High school	18	19	17	19
Higher education	47	47	46	46
Advanced degree	18	19	19	20
Any smoke exposure (%)	48	50	46	49
Smoking status (%)
Never	51	49	48	46
Former	36	38	42	44
Current	13	13	10	10
Pack-years of smoking (%)
0	52	50	54	52
≤ 10	19	19	16	15
> 10 and ≤ 20	10	10	9	9
> 20	20	21	21	23
Residential stability (years)
≥ 10	69	75	68	75
≥ 20	45	52	44	51
Study site (%)
Winston-Salem, NC	15	17	13	15
New York, NY	18	16	23	19
Baltimore, MD	14	16	11	14
St. Paul, MN	15	17	13	15
Chicago, IL	18	18	18	19
Los Angeles, CA	20	16	23	17
Air pollution
PM_2.5_ (μg/m^3^)	16.3 ± 3.7	22.0 ± 5.0	14.2 ± 2.4	22.2 ± 5.0
PM_10_ (μg/m^3^)	NA	34.3 ± 7.7	NA	34.7 ± 7.7
NO_x_ (ppb)	48.3 ± 25.2	NA	41.1 ± 21.1	NA
NA, not applicable. ^***a***^Air flow restriction defined as an FEV_1_/FVC and FEV_1_ less than the lower limit of normal (LLN).				

**Figure 1:**
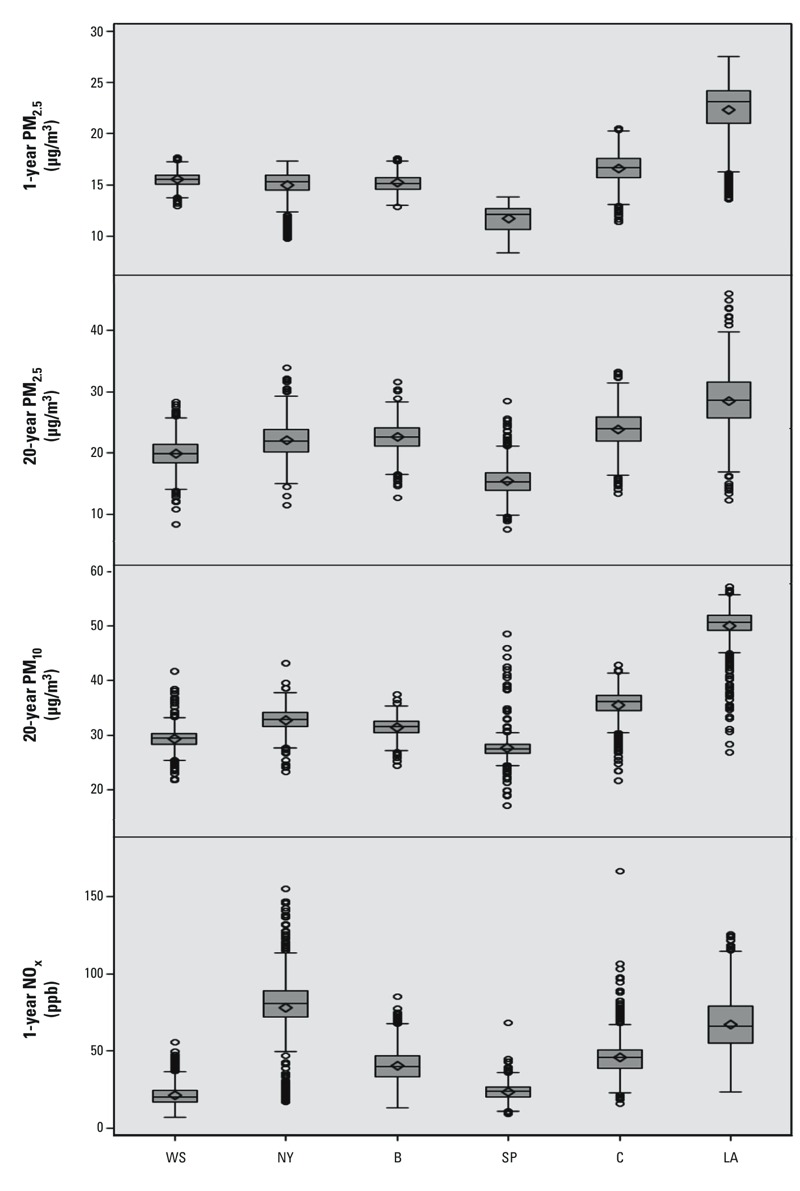
Distribution of individual-level estimates of long-term PM_2.5_, PM_10_, and NO_x_ concentrations at participant residences by city and averaging period. Abbreviations: B, Baltimore; C, Chicago; LA, Los Angeles; NY, New York; SP, St. Paul; WS, Winston-Salem. Scales vary by plot. Boxes extend from the 25th to the 75th percentile, horizontal bars represent the median, diamonds represent the means, whiskers extend 1.5 times the length of the interquartile range above and below the 75th and 25th percentiles, respectively, and outliers are represented as points.

Long-term estimates of each air pollutant are presented in Table 1. Concentrations declined over time, such that the 20-year averages of PM_2.5_ were consistently higher than the more recent 1-year average levels. Spatial contrasts in PM_2.5_ were consistent over time, however, with the highest concentrations in Los Angeles and the lowest concentrations in St. Paul (Figure 1). PM_10_ followed similar spatial patterns and was highly correlated with PM_2.5_ in the overall data (ρ: 0.7–0.9) but weakly correlated after stratification by metropolitan area (average ρ: 0.1–0.3). NO_x_ had lower correlations with PM_10_ and PM_2.5_ (overall ρ: 0.5–0.6; area-specific ρ: 0.1–0.3). Similar concentrations of PM_2.5_ and NO_x_ were found between the 1-year and 20-year cohorts except for New York and Los Angeles, where additional study subjects reduced the mean concentrations slightly and increased the overall variability (results not shown).

Table 2 presents relationships between percent emphysema with the different air pollutants and averaging times examined. Without adjustment for study site, higher levels of all pollutants were associated with greater percent emphysema. For example, 5 μg/m^3^ greater PM_2.5_ and 25 ppb higher NO_x_ concentrations over the year preceding the clinical visit were associated with 0.6 [95% confidence interval (CI): 0.1, 1.2%] and 0.5 (95% CI: 0.1, 0.9%) higher average percent emphysema. However, after adjustment for study site the associations were –0.6% (95% CI: –1.5, 0.3%) for PM_2.5_ and –0.5% (95% CI: –1.1, 0.02%) for NO_x_.

**Table 2:** Associations (95% CIs, *p*-values) between long-term concentrations of pollutants and percent emphysema on CT.

Model	1-year average		20-year average	
	PM_2.5_ (*n *= 6,515)	NO_x_ (*n *= 6,515)	PM_2.5_ (*n *= 4,813)	PM_10_ (*n *= 4,813)
Minimal control (demographics)	0.4 (–0.1, 0.8)	0.3 (0.0, 0.6)	1.0 (0.7, 1.4)	0.4 (0.1, 0.6)
Moderate control (risk factors)	0.6 (0.1, 1.2)	0.5 (0.1, 0.9)	1.0 (0.6, 1.4)	0.4 (0.1, 0.7)
Full control (site adjusted)	–0.6 (–1.5, 0.3)	–0.5 (–1.1, 0.0)	0.2 (–0.3, 0.7)	–0.5 (–1.2, 0.2)
Associations were scaled to 5 μg/m^3^ for PM and 25 ppb for NO_x_. Minimal control models were adjusted for age, race/ethnicity, and sex. Moderate control models added height, body mass index, education, household size, birth location, smoking, examination, scanner, and scanner by body size. Full control models incorporated site adjustment using a fixed effect.				

**Figure 2:**
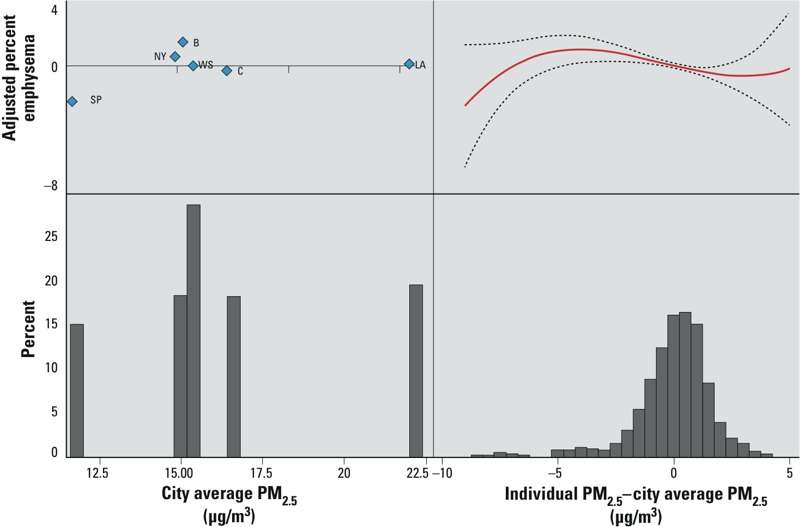
Adjusted relationships between percent emphysema and 1-year PM_2.5_ concentrations expressed as between-site (city average) and within-site (individual concentration–city average) gradients. The left panel illustrates adjusted city mean emphysema vs. city average PM_2.5_ concentrations. This reflects the information provided by between-city contrasts. The right panel illustrates the continuous dose–response relationship (in red; 95% CI in dashed lines) between adjusted percent emphysema vs. within-city contrasts in exposures. In both panels, the bottom of the figure represents a frequency distribution of exposures. Abbreviations: B, Baltimore; C, Chicago; LA, Los Angeles; NY, New York; SP, St. Paul; WS, Winston-Salem. All models were adjusted for age, race/ethnicity, sex, height, body mass index, education, household size, birth location, smoking, examination, scanner, and multiple detector computed  tomography scanner by body size.

**Figure 3:**
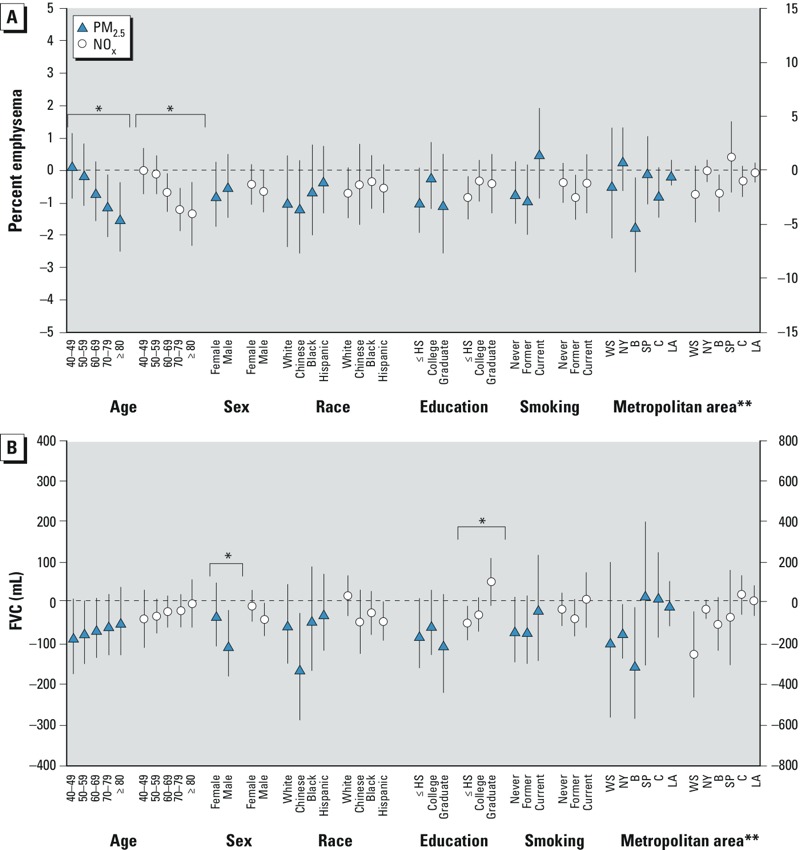
Associations (95% CIs) between 1-year average PM_2.5_ and NOx concentrations with percent emphysema and FVC by selected personal factors. Models were adjusted for age, race/ethnicity, sex, height, body mass index, education, household size, birth location, smoking, examination, and site. Percent emphysema was further adjusted for scanner and MDCT scanner by body size. Lung function was further adjusted for detailed smoke exposures, workplace exposures, and hay fever. Abbreviations: B, Baltimore; C, Chicago; HS, high school; LA, Los Angeles; NY, New York; SP, St. Paul; WS, Winston-Salem. *Significant effect modification (*F*-test *p*-value < 0.05). **Metropolitan area results are presented on secondary (right-hand) axis.

Closer inspection of the data suggested that associations observed before adjustment for study site were strongly influenced by statistically significantly lower mean percent emphysema in St. Paul (see Supplemental Material, Table S1), where air pollution levels were also lowest. The importance of between-city contrasts can be visualized in Figure 2, where the average percent emphysema for each city after controlling for other risk factors is plotted against the city-average 1-year PM_2.5_ concentrations. In fact, positive associations between percent emphysema and pollution levels were not observed in models excluding St. Paul (results not shown) or for within-city contrasts in any of the study sites (Figure 3).

Decreased lung function was consistently observed with higher concentrations of PM_2.5_ and NO_x_ with and without adjustment for site, although many of the associations did not meet statistical significance (Table 3; see also Supplemental Material, Figure S2). The relationships of the greatest magnitude were between the 1-year average PM_2.5_ concentrations and FVC with –54 mL (95% CI: –91, –18 mL) and –59 mL (95% CI: –132, 13 mL) lower FVC per 5 μg/m^3^ before and after control for site, respectively. The 1-year PM_2.5_ concentration was also more strongly associated with FEV_1_ than 20-year PM_2.5_ concentrations, with –24 mL (95% CI: –54, 6 mL) and –20 mL (95% CI: –80, 41 mL) lower FEV_1_ per 5 μg/m^3^ before and after control for site, respectively. Higher PM_2.5_ concentrations (5 μg/m^3^) over the previous day were associated with lower FEV_1_ (–5 mL; 95% CI: –13, 4 mL) and FVC (–3 mL; 95% CI: –13, 7 mL) though these could not be distinguished from no association. Associations between all lung function metrics and PM_10_ were positive but with wide confidence intervals. No consistent associations were observed with the ratio of FEV_1_/FVC or airflow limitation.

**Table 3:** Associations (95% CIs) between pollutants and lung function.

Model	1-year average		20-year average	
	PM_2.5_ (*n *= 3,791)	NO_x_ (*n *= 3,791)	PM_2.5_ (*n *= 2,811)	PM_10_ (*n *= 2,811)
Difference in mean FEV_1_ (mL)
Minimal control (demographics)	–27 (–58, 4)	–22 (–40, –4)	–4 (–21, 13)	13 (1, 24)
Moderate control (risk factors)	–24 (–54, 6)	–12 (–30, 7)	–15 (–31, 2)	6 (–5, 18)
Full control (site adjusted)	–20 (–80, 41)	–4 (–33, 25)	–13 (–37, 11)	1 (–30, 32)
Difference in mean FVC (mL)
Minimal control (demographics)	–64 (–101, –26)	–20 (–42, 2)	–9 (–29, 12)	12 (–2, 26)
Moderate control (risk factors)	–54 (–91, –18)	–9 (–31, 14)	–19 (–39, 0)	6 (–8, 20)
Full control (site adjusted)	–59 (–132, 13)	–21 (–55, 14)	–6 (–35, 22)	19 (–29, 45)
Difference in mean FEV_1_/FVC (%)
Minimal control (demographics)	0.6 (0.0, 1.1)	–0.3 (–0.8, 0.0)	0.1 (–0.2, 0.4)	0.1 (–0.1, 0.3)
Moderate control (risk factors)	0.4 (–0.2, 1.0)	–0.3 (–0.5, 0.0)	0.0 (–0.3, 0.3)	0.1 (–0.2, 0.3)
Full control (site adjusted)	0.2 (–0.9, 1.3)	0.3 (–0.3, 0.8)	–0.3 (–0.7, 0.2)	0.3 (–0.8, 0.4)
Odds of airflow limitation
Minimal control (demographics)	1.2 (0.9, 1.6)	1.3 (1.1, 1.5)	1.1 (0.9, 1.3)	1.0 (0.9, 1.1)
Moderate control (risk factors)	1.2 (0.9, 1.6)	1.3 (1.0, 1.5)	1.2 (1.0, 1.4)	1.1 (0.9, 1.2)
Full control (site adjusted)	0.9 (0.5, 1.7)	1.1 (0.8, 1.4)	1.1 (0.8, 1.5)	1.1 (0.8, 1.6)
Associations were scaled to 5 μg/m^3^ for PM and 25 ppb for NO_x_. Minimal control models included age, race/ethnicity, and sex. Moderate control models added height, body mass index, education, household size, birth location, smoking, examination, detailed smoke exposures, workplace exposures, and hay fever. Full control included site adjustment using a fixed effect.				

In secondary analyses, we found limited evidence of effect modification of associations by personal characteristics (Figure 3). The most consistent findings across pollutants and outcomes were increasingly negative associations between air pollution and percent emphysema and increasingly positive associations with lung function measures among persons of greater age in models adjusting for study site. There was also some evidence of significant effect modification of the relationship between NO_x_ and FVC as well as FEV_1_ (results not shown) by sex and education, but the same was not true for PM_2.5_. Other sensitivity analyses indicated that all results were qualitatively robust (similar magnitude, direction, and significance) to using an alternate definition of airflow limitation and restricting to individuals who had not moved in the previous 10 years (results not shown). Significant positive associations were also demonstrated between percent emphysema defined using a –950 HU threshold with the 1-year average of NO_x_ and 20-year average of PM_2.5_ before adjustment for study site, though less consistent findings were found with the other pollutants. All associations with percent emphysema defined by –950 HU had similar directionality and significance after controlling for study site (results not shown).

## Discussion

In this large, multi-center study, we found weak evidence of an association between long-term exposures to air pollution and emphysema. Higher long-term PM_2.5_, PM_10_, and NO_x_ concentrations between study sites were associated with greater percent emphysema, though these findings were driven by differences between study sites and were not replicated for within-site exposure contrasts. Suggestive but imprecise associations were also identified between air pollution and lung function, with lower FEV_1_ and FVC observed among persons with higher long-term levels of PM_2.5_ and NO_x_.

This research is unique in its use of percent emphysema on CT scan to study associations between air pollution exposures and respiratory health in a large cohort. CT scans may be a valuable tool for air pollution epidemiology studies because they allow for quantification of early changes in lung structure, as opposed to lung function, which is assessed by traditional spirometry testing. This may lead to important contributions because a recent review of the associations between air pollution and COPD (Schikowski et al. 2014) discussed the limitations of existing studies in their ability to characterize subclinical phenotypes and progression of COPD. Although careful consideration must be made given the additional cost and radiation exposure to participants, albeit small, percent emphysema may also have clinical importance because it has been linked with increased risks of mortality in several, though not all, studies (Dawkins et al. 2003; Haruna et al. 2010; Johannessen et al. 2013; Martinez et al. 2006; Sverzellati et al. 2012).

Although little is known of air pollution’s impacts on emphysema, past research generally supports a link between the inhalation of ambient pollutants and adverse impacts on the pulmonary system (Kelly and Fussell 2011). Biologically, this is hypothesized to occur via several interconnected mechanisms including pulmonary oxidative stress and inflammation (Adar et al. 2007; Budinger et al. 2011; Happo et al. 2010; Stringer and Kobzik 1998), alterations in airway ciliary activity (Calderón-Garcidueñas et al. 2001), as well as enhanced susceptibility to respiratory infections (Stern et al. 2013), which can ultimately lead to long-term damage to the lungs including loss of alveolar tissue (i.e., emphysema). Although the larger inhaled particles of tobacco smoke or ambient PM are deposited higher in the airways and likely result in a more classically bronchitic phenotype, PM_2.5_ deposits more heavily in the alveoli, likely resulting in more parenchymal rather than airway damage (U.S. Environmental Protection Agency 2009).

Consistent with the toxicological literature, epidemiology studies similarly show evidence of increased respiratory symptoms and hospitalizations with air pollution exposure (Bayer-Oglesby et al. 2006; Brauer et al. 2007; Dominici et al. 2006; Martins et al. 2002) as well as evidence of slowed lung growth among cohorts of children followed over time in several different countries (Gauderman et al. 2004; Horak et al. 2002; Mölter et al. 2013). The SAPALDIA study (Swiss Study on Air Pollution and Lung Diseases in Adults) similarly demonstrated slower age-related declines in FEV_1_ with larger reductions in pollution over time in approximately 10,000 Swiss adults (Downs et al. 2007), though no association was reported between NO_2_ and FEV_1_ decline among 2,644 British adults (Pujades-Rodríguez et al. 2009). Higher long-term concentrations of air pollutants, including particles and traffic-related pollutants, have also been associated with increased odds of COPD in Germany (Schikowski et al. 2005) and risk of incident COPD hospitalizations in Denmark and Canada (Andersen et al. 2011; Gan et al. 2013). A smaller study of approximately 400 German women further reported lower prevalent COPD with larger reductions in PM_10_ over time (Schikowski et al. 2010). Occupational settings have shown linkages between particulate exposures, emphysema, and COPD even after control for cigarette smoking (Coggon and Newman Taylor 1998; Diaz-Guzman et al. 2012; Green et al. 1998). Although one analysis of long-term exposure to PM_2.5_ linked higher concentrations with lower risk of COPD death in the United States, this work relied on death certificates for outcome ascertainment, and it was hypothesized that this unexpected apparent protective relationship may have been an artifact of competing risks, because pneumonia and cardiovascular events were positively associated with air pollution (Pope et al. 2004).

In this study, we also found consistent evidence of inverse associations between air pollution and emphysema among the oldest participants (70–79 and ≥ 80 years) for both PM_2.5_ and NO_x_ as well as weaker associations between pollution and lung function among the oldest participants. These unexpected findings can likely be explained by the unique population of MESA, which recruited older adults without clinical cardiovascular disease at baseline. Given that air pollution has also been linked to cardiovascular disease (Brook et al. 2010), our findings of increasingly negative associations with greater age may simply reflect the selection of older individuals in the study who are healthier and less susceptible to air pollution than the general population.

Within MESA, exposure and outcomes varied substantially between study sites, and these differences were especially influential in models for emphysema. As a result, our results for percent emphysema but not lung function were sensitive to adjustment for study site. Importantly, our results remained largely insensitive to control for personal-level socioeconomic status including education, household size, and a wealth index. Nevertheless, there remains the possibility for residual confounding by unmeasured factors. Regional differences may have played an important role: A detailed investigation of our findings suggests that our overall results for percent emphysema were strongly influenced by data from St. Paul, which had low levels of COPD and low levels of pollution. Interestingly, scanner technology cannot explain these differences because the same scanner used in St. Paul was also used at another study site, and the differences in mean percent emphysema were found even after control for scanner. Although control for study site is likely warranted, even if only to properly estimate our standard errors, including such control reduced the exposure variability given the large contrasts in exposure between locations. Thus, there may be power issues in detecting differences within-city.

An additional possible weakness of this work is that percent emphysema was measured using cardiac scans, which do not include the lung apices and hence may have underestimated the degree of emphysema compared with a full-lung scan. However percent emphysema measurements on MESA cardiac scans have been previously validated against full-lung scans (Hoffman et al. 2009) and health outcomes (Barr et al. 2010, 2012).

A major strength of this study was that we used a well-defined cohort with rich estimates of PM and traffic-related pollutants in outdoor air that capture both spatial and temporal trends. Individual-level 1-year average concentrations were derived using data from intensive monitoring campaigns in participants’ comunities and homes. These estimates were complemented by 20-year estimates, which inform us of long-term exposures over a participant’s long-term residential history, although they have substantially less precision for fine-scale spatial variability. Generally consistent findings were observed for the 1-year and 20-year estimates. In addition, our results were robust among persons with long-term (> 10 years) residential stability.

In summary, this cross-sectional analysis of a large, multi-center, population-based cohort found some suggestive evidence to support the hypothesis that higher long-term air pollution exposures are associated with emphysema. Because results were dominated by contrasts between study sites, however, future work is required to confirm our findings.

## Supplemental Material

(460 KB) PDF
