# Supplementary material for: Air Pollution and Percent Emphysema Identified by Computed Tomography in the Multi-Ethnic Study of Atherosclerosis
Source: Environ Health Perspect. 2014 Oct 10;123(2):144–51. doi: 10.1289/ehp.1307951 (PMC4314244; doi:10.1289/ehp.1307951)
Supplement: (460 KB) PDF [file ehp.1307951.s001.508.pdf]

Environmental Health Perspectives doi:10.1289/ehp.1307951

Supplemental Material

Air Pollution and Percent Emphysema Identified by Computed Tomography in the Multi-Ethnic Study of Atherosclerosis

Sara D. Adar, Joel D. Kaufman, Ana V. Diez-Roux, Eric A. Hoffman, Jennifer D'Souza, Karen D. Hinckley Stukovsky, Stephen S. Rich, Jerome I. Rotter, Xiuqing Guo, Leslie J. Raffel, Paul D. Sampson, Assaf P. Oron, Trivellore Raghunathan, and R. Graham Barr

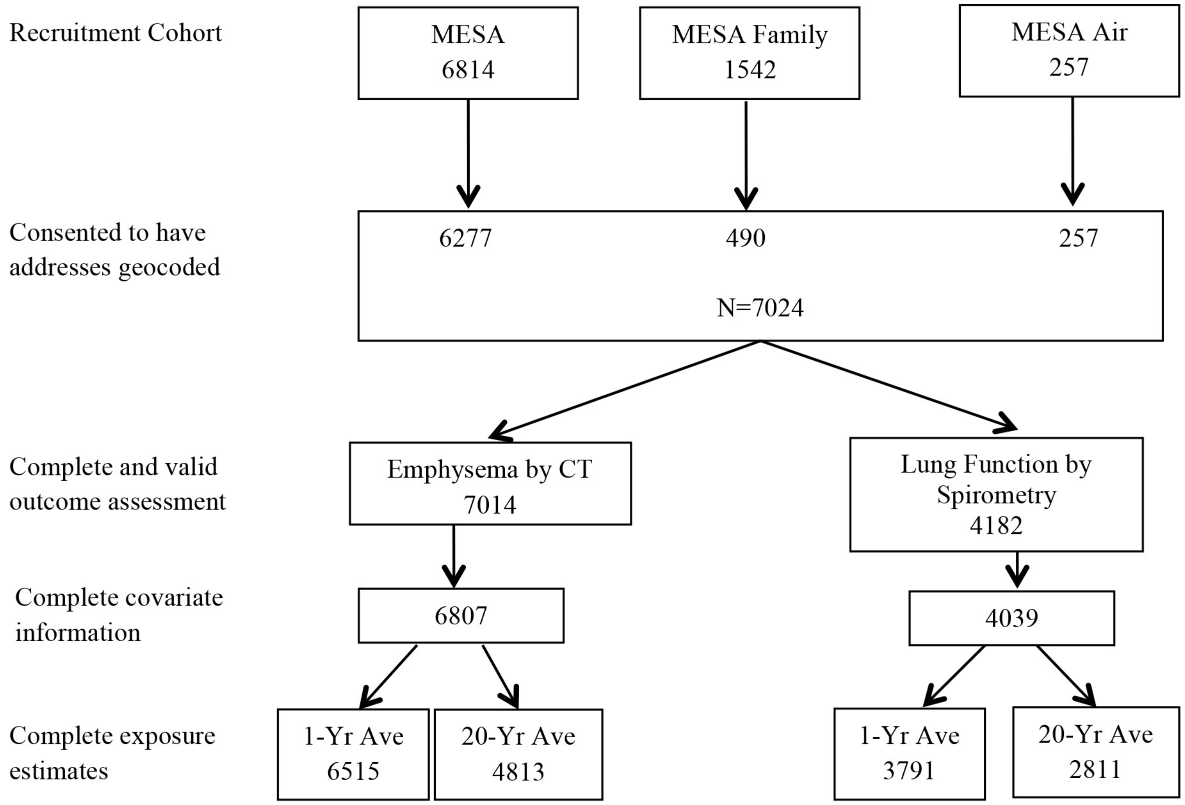

Figure S1. Sample size for various endpoints and exposures.

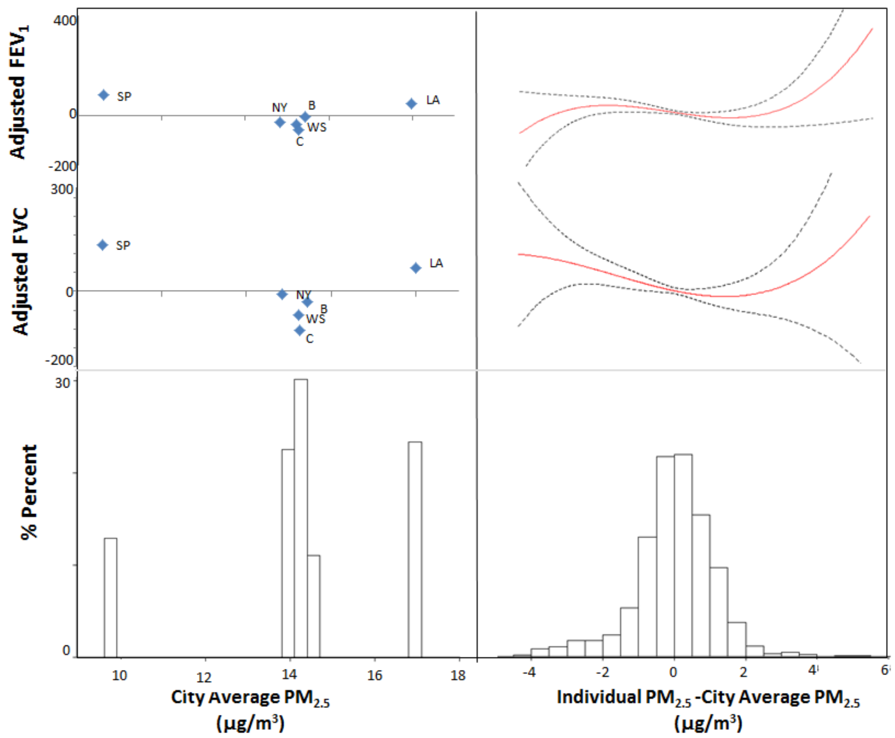

Figure S2. Adjusted lung function vs. $PM_{2.5}$ concentrations expressed as between-site (city average) and within-site (individual concentration - city average) gradients. The left panel illustrates adjusted city mean FEV_1 and FVC vs. city average $PM_{2.5}$ concentrations. This reflects the information provided by between-city contrasts. SP=St Paul, B=Baltimore, NY=New York, WS=Winston Salem, C=Chicago, LA=Los Angeles. The right panel illustrates the continuous dose-response relationship (in red, 95% CI in dashed lines) between adjusted FEV_1 and FVC vs. within-city contrasts in exposures. Overall model included age, race/ethnicity, gender, height, body mass index, education, number of people in household, birth location, smoking information, exam number, detailed smoke exposures, workplace exposures, and history of hay fever. The site-controlled model included an additional fixed effect for study site. In both panels, the bottom of the figure represents a frequency distribution of exposures.

Table S1. Differences in percent emphysema by study site compared to the reference group of Los Angeles in fully adjusted models.

Metropolitan area	Percent emphysema
Winston-Salem, NC	-0.1 (-3.1, 2.9)
New York City, NY	-0.6 (-2.2, 1.0)
Baltimore, MD	-1.6 (-3.7, 0.6)
St.Paul, MN	-7.1 (-9.5, -4.7)
Chicago, IL	-0.8 (-2.2, 0.6)
Los Angeles, CA (referent group)	Referent

Note: Overall model included age, race/ethnicity, gender, height, body mass index, education, number of people in household, birth location, smoking information, exam number, detailed smoke exposures, workplace exposures, history of hay fever, and fine particulate air pollution.
